# Prevalence and associated risk factors of anaemia among women attending antenatal and post-natal clinics at a public health facility in Ghana

**DOI:** 10.1186/s40795-019-0303-x

**Published:** 2019-09-23

**Authors:** Philip Kofie, Elvis E. Tarkang, Emmanuel Manu, Hubert Amu, Martin Amogre Ayanore, Fortress Yayra Aku, Joyce Komesuor, Martin Adjuik, Fred Binka, Margaret Kweku

**Affiliations:** 1 Department of Epidemiology and Biostatistics, School of Public Health, University of Health and Allied Sciences, PMB 31, Ho, Volta Region Ghana; 2 Department of Population and Behavioural Sciences, School of Public Health, University of Health and Allied Sciences, PMB 31, Ho, Volta Region Ghana; 3 Department of Family and Community Health, University of Health and Allied Sciences, PMB 31, Ho, Volta Region Ghana

**Keywords:** Anaemia, Pregnant women, Post-partum mothers, Antenatal care, Post-natal care, Hohoe municipality, Hohoe municipal hospital, Ghana

## Abstract

**Background:**

Anaemia among pregnant women and post-partum mothers is a public health challenge in Ghana, especially in the Volta Region. While literature abounds on anaemia among pregnant women, the same cannot be said for anaemia among post-partum mothers in the region. This study, therefore, examined the prevalence and associated risk factors of anaemia among women attending antenatal care and post-natal care.

**Methods:**

This descriptive cross-sectional survey recruited 409 pregnant women and 194 post-natal mothers attending antenatal and post-natal care, at the Hohoe Municipal Hospital. Background characteristics were collected using a semi-structured questionnaire, blood samples were analysed for the presence of anaemia and malaria parasitaemia and folders were reviewed for estimated blood loss.

**Results:**

We found the prevalence of anaemia among pregnant women and post-partum mothers to be 33 and 16% respectively. Higher malaria parasitaemia (2%) was found in pregnant women compared with postpartum mothers (1%). We found that 4% of post-partum mothers had abnormal blood loss (301mls-500mls) whereas 5% of them had postpartum haemorrhage (>500mls) during child birth. A univariate logistics regression of anaemia status on some risk factors in pregnant women showed no significant association between anaemia and any of the risk factors. Among post-partum mothers, only mothers’ age was statistically significant in the univariate analysis [COR = 0.27 (95% CI:0.103, 0.72);0.008]. Mothers aged 20–29 were 73% less likely to be anaemic.

**Conclusion:**

The prevalence of anaemia among pregnant women found in this study points to a situation of moderate public health problem according to WHO cut-off values for the public health significance of anaemia. Strategies should therefore be put in place to encourage thorough health education and promotion programmes among both pregnant and post-partum women.

## Background

Anaemia, referred to as low level of haemoglobin (Hb) in the blood, is a global public health problem that affects low, middle, and high-income countries, with adverse effects on the health of populations [1]. Anaemia is defined as Hb level lower than 11.0 g/dl (Hb < 11.0 g/dl) in pregnant women [2], and Hb level lower than 10.0 g/dl (Hb < 10.0 g/dl) in postpartum mothers [3]. Though the condition affects males, pregnant and non-pregnant women as well as children are most vulnerable [4, 5].

Anaemia is multifactorial in aetiology but mainly caused by iron-deficiency [6, 7]. Anaemia can be dangerous to the health of both a pregnant woman and her baby, if left untreated [8] as it increases the risk of maternal and child mortality and also has a significant negative effect on both the cognitive and physical development of the child [4, 9].

Global anaemia prevalence is estimated by the WHO to be 38% in pregnant women and 29% in all women of reproductive age [1]. According to WHO cut of points for significance of anaemia, prevalence of ≥40% is considered a severe public health problem [1]. In Africa, a 2013 cross-sectional study conducted among 384 pregnant women in Northwest Ethiopia found the prevalence of anaemia to be 22% [10]. A 2016 study conducted by Bekele, Tilahun and Mekuria [11] among 332 pregnant women in the same country, however, found anaemia prevalence to be 33%, an indication that the problem was on the ascendency. Furthermore, a cross-sectional secondary data analysis on anaemia prevalence among post-partum mothers in the same country found 22% prevalence rate [12], lower than the prevalence rates among pregnant women [10, 11].

In Ghana, a report by the Family Health Division (FHD) of the Ghana Health Service (GHS), showed that anaemia among pregnant women at first antenatal clinic visit marginally increased by 1% in the year 2015 as compared to the previous year [13]. The report further stated that the prevalence of anaemia in pregnant women at 36 weeks of pregnancy also increased marginally from the year 2015 to 2016. The Volta Region has been identified as the region with the highest prevalence (49%) of anaemia among women in their reproductive age (15–49 years) in the country [14]. As such, it is the region with the highest proportion of antenatal clients having anaemia over the period of 2014 to 2016, with prevalence rates of 46, 46, and 47% in 2014, 2015 and 2016 respectively [13].

The FHD reported anaemia in the region at the time of antenatal clinic registration and at 36 weeks of pregnancy to be 46 and 32% respectively in 2014; 46 and 26% respectively in 2015; and 47 and 27% respectively in 2016 [13]. Although there is provision of iron and folic acid to postnatal mothers from the day of delivery up to the sixth week in the country [13], anaemia remains the leading cause of hospital admissions and maternal deaths in the Volta Region [15, 16, 17]. In the Hohoe Municipality where the present study was conducted, the prevalence of anaemia in pregnant women attending antenatal care was reported to be 60.3% [18], with 64% among new registrants and 58% among those with multiple visits. This prevalence is higher than the regional prevalence of 49%.

Despite the available evidence on anaemia and its consequences, there has not been any study on postpartum anaemia in the Volta Region of Ghana. Postpartum period, characterized by some physiological losses due to pregnancy and labour is a very critical period which needs a lot of attention. The paucity of information on anaemia status of this vulnerable group in the region possess as a weakness of health system as no or little information is available to guide health professionals in ensuring good health of postpartum mothers. We, therefore, examined the prevalence and associated risk factors of anaemia among women attending antenatal and post-natal clinics at the Hohoe Municipal Hospital so that a holistic approach in addressing maternal anaemia in the municipality, the region, and the country as a whole could be adopted.

## Methods

### Setting

The Hohoe Municipality is one of the twenty-five administrative districts/municipalities in the Volta Region [19]. The Municipality lies within the middle zone of the region and shares boarders on the East with the Republic of Togo, on the Southwest with Kpando Municipality, Northwest with Biakoye District, on the North with Jasikan District, and on the South with Afadzato South District [19]. The Municipality consists of 102 communities with a population of 167,016 people and a population density of 196.0 persons per square kilometres [19].

### Study design

This descriptive cross-sectional study recruited 409 pregnant women (6 weeks to 36 weeks of gestation) and 194 postnatal mothers (6 weeks post) of ANC and PNC centres of Hohoe Municipal Hospital for the period March 2017.

#### Procedures

We estimated the sample sizes for pregnant women using the regional prevalence of 47% [13] and 14% for post-partum mothers [20], using the Cochrane formula [21]. Assuming z-statistic for 95% level of confidence and a 5% margin of error, the appropriate minimum sample size was estimated for the study.

Adjusting for a non-response rate of 5%, a total sample size of 402 was reached for pregnant women and 194 for post-partum mothers.

During data collection, thirty (30) pregnant women were randomly selected each day by balloting without replacement method. The balloting method allowed consented participants to either pick “Yes” or “No” of folded pieces that were placed in a container and thoroughly shaken to ensure randomization. Data was collected from participants who picked “Yes”. This was repeated until the desired sample size was attained. The same sampling procedure was done with regards to the postnatal mothers. Data were collected at PNC clinic and post-delivery wards of the Hospital.

With the aid of trained research assistants, data on socio-demographic characteristics and risk factors associated with anaemia (independent variables) in both pregnant and postpartum mothers were collected using a pre-tested semi-structured questionnaire. Haemoglobin concentration (Hb) were determined by finger-pricked blood test samples of participants using URIT-12 Haemoglobin photometer (URIT Medical Electronics Co., LTD, China). Anaemia was defined as Hb level lower than 11.0 g/dl (Hb < 11.0 g/dl) in pregnant women [2] and Hb level lower than 10.0 g/dl in postpartum mothers (Hb < 10.0 g/dl) [3]. Capillary blood sampling from the finger was used because it provides a reliable and suitable alternative for sampling blood in the clinical and field settings [22–24].

Estimated blood loss data of postpartum mothers were obtained from the maternal delivery records. Blood loss volume of ≤300 ml was considered normal and > 300 ml–500 ml was considered abnormal. Blood loss volume (>500mls) was considered postpartum haemorrhage (PPH) [21].

Parasitaemia in blood samples were detected using standardized blood film and staining procedures [25]. Three drops of blood were placed on a clean, dust free and dry frosted microscopic slide for thick blood film. Also a drop of blood was placed on the side of the thick film for the preparation of a thin film. A unique Identification number (ID) for each participant and date were written on each slide for easy identification. The slides were air dried and packed into slide boxes and transported to the SPH, UHAS laboratory. The dried slides were stained with 1% Giemsa stain for about 25–30 min. Buffered water (pH = 7) was used to rinse the stained slides. The prepared slides were examined under oil immersion with a light microscope (ocular magnification × 100). The thick film was used for the quantification of the malaria parasites while the thin film was used for identifying the malaria species. Parasite densities were estimated by counting the number of parasites per 200 white blood cells (WBCs) in a thick film by two microscopists. Counts of gametocyte were taken against 500 white blood cells in determining gametocyte density per microliter of blood.

Light microscope was used to read the slides, a sample was considered negative only after 200 high power fields have been read. Parasite counts were converted to parasite per μ1, with the assumption that there is an average of 8000 leucocytes per μ1 of blood. In cases where there was a 50% discrepancy between parasite counts or when there was a discrepancy qualitatively (negative versus positive), a third microscopist read the slide and his reading was final and was used in the analysis of parasite density. All slides were stored in appropriately labelled slide boxes and kept at the laboratory. As part of the quality control monitoring, randomly selected stained slides from each batch of slides were given to an independent microscopist at the Municipal hospital for the determination of the sensitivity and specificity of the readers. Hb readings were quality controlled by trained laboratory scientists from the School of Public Health (SPH) of the University of Health and Allied Sciences (UHAS), research laboratory throughout the study period.

### Ethical issues

This study was conducted in accordance with accepted principles on ethics in human experimentation and international conference on Harmonization/Good Clinical Practice (ICH/GCP). Ethical approval for the study was obtained from the Ethics Review Committee (ERC) of the University of Health and Allied Sciences (UHAS) with Ethical Approval number UHAS-ERC A.6 [6] 17–18. Permission was sought from the Hohoe Municipal Hospital before the commencement of the study. Written informed consent was obtained from participants on standard consent form before they were included in the study.

### Data analysis

The data were entered into EpiData version 3.0 and exported to Stata version 14.1 for analysis. Descriptive statistics, frequencies and percentages were used for categorical variables. Normality was determined for continuous variables such Age, Hb, gravidity, parity and family size. Mean ± SD was determined for continuous variables using t-test. Chi-square test was used to determine association between independent variables (socio-demographic characteristics and risk factors) and the dependent variable (anaemia status). A univariate logistic regression was used to determine the strength of the association between the independent and dependent variable. The dependent variable considered in the univariate logistic regression was anaemia status. However, a multivariable logistic regression could not be used because only one variable in the univariate logistic regression showed a statistically significant association with the dependent variable (Anaemia status). Therefore, there was no need to use a multivariable logistic regression. A *p*-value less than 0.05 was considered statistically significant at 95% Confidence Interval (CI).

## Results

### Socio-demographic characteristics of respondents

Table 1 shows the background characteristics of the respondents, for both ANC and PNC attendees. The continuous variables ag and Hb were normally distributed. Out of the 409 pregnant women surveyed, majority (49%) of them were aged 20–29 years. The mean age of the pregnant women was 28 ± 7 years. Most, (83%) of the pregnant women were married and had at least Junior Secondary education (65%). With regards to occupation, about two-fifth of the pregnant women (38%) were involved in trading at the time of the study. Majority (71%) of them were Ewes, with Christianity being the dominant religion (85%). More than half (54%) of the pregnant women were at least, pregnant for the second time (Gravida 2) in their lives at the time of the survey. A little less than half of (41%) respondents had at least one or two children. More than half (77%) of pregnant women were in their 3rd trimester.

**Table 1:** Background characteristics of women attending ANC and PNC clinics [*N* = 603]

Characteristic	ANC [*N* = 409]	PNC [*N* = 194]
Mean Age (SD)	28 ± 6	27 ± 7
Age group (in years)		
< 20	49 (12)	33 (17)
20–29	208 (51)	96 (49)
30–39	138 (34)	58 (29)
40>	14 (3)	10 (5)
Marital Status		
Single	68 (17)	41 (21)
Married	341 (83)	156 (79)
Educational Level		
None	30 (7)	13 (7)
Primary	71 (17)	37 (19)
Secondary (JHS/SHS)	267 (65)	126 (64)
Tertiary	41 (10)	21 (10)
Occupation		
Unemployed	102 (25)	53 (27)
Artisan	103 (25)	49 (25)
Civil servant	37 (9)	17 (9)
Farming	15 (3)	15 (7)
Trading	152 (38)	63 (32)
Religion		
Christianity	346 (85)	167 (85)
Muslim	63 (15)	30 (15)
Tribe		
Ewe	291 (71)	148 (75)
Others	118 (29)	49 (25)
Mean Haemoglobin level (g/dl)	11. 78 ± 1.93	11.82 ± 2.48
Gravidity		
1–2	219 (54)	116 (59)
3–5	168 (41)	66 (34)
6≤	22 (5)	15 (7)
Parity		
None (Nulliparous)	144 (35)	–
1–2	168 (41)	118 (60)
3+	97 (24)	79 (40)
Gestational age		
1st trimester	58 (14)	–
2nd trimester	37 (9)	–
3rd trimester	314 (77)	–

### Prevalence of anaemia and malaria among pregnant and postpartum women attending ANC and PNC clinics

**Fig. 1:**
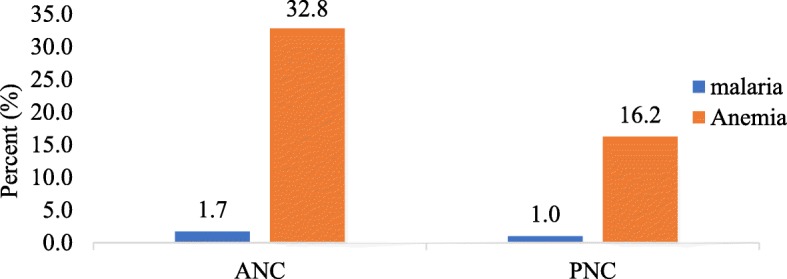
Prevalence of malaria and anaemia in pregnant and post-partum women

Out of the total number (409) of pregnant women whose Hb levels were measured, 33% them were found to be anaemic (Hb < 11.0 g/dl) with a mean Hb of 9.72 ± 0.97. Out of the mothers (194) attending PNC, 16% of them were anaemic (Hb < 10 g/dl). The mean Hb recorded for the postpartum mothers was 7.76 ± 1.68 (Fig. 1). The prevalence of malaria parasitaemia by microscopy was 2 and 1% for pregnant women and postpartum mothers respectively (Fig. 1).

### Prevalence of anaemia and malaria among pregnant women by gestational period

**Fig. 2:**
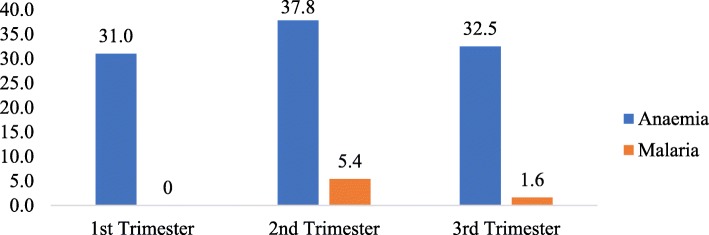
Prevalence of anaemia and malaria among pregnant women by gestational period

The prevalence of anaemia among pregnant women by gestational period was highest (38%) among women in their 2nd trimester and lowest (31%) among those in their 1st trimester (Fig. 2). Malaria prevalence followed similar pattern as anaemia, 5% for pregnant women in their 2nd trimester, 1.6% in the 3rd trimester and none in the 1st trimester.

### Estimated blood loss of postpartum mothers

**Fig. 3:**
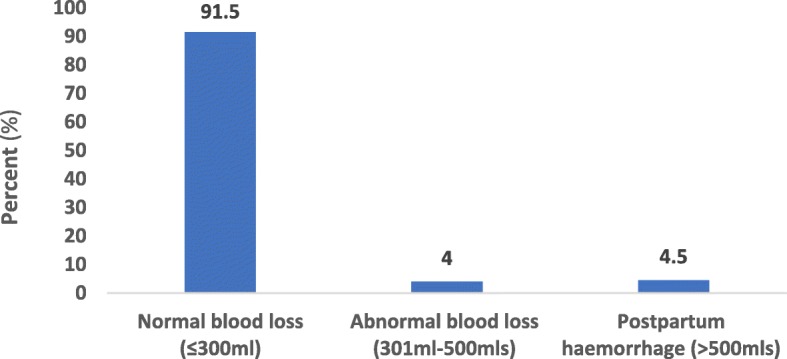
Post-partum blood loss by mothers who delivered at the Hohoe Municipal Hospital

Out of the total number (177) of mothers whose records were retrieved for the estimation of blood loss (Fig. 3), majority (92%) had normal blood losses (≤300 ml), (4%) of them had abnormal blood loss (301 ml-500mls) and (5%) had postpartum haemorrhage (>500mls).

### Association between Anaemia and some risk factors among pregnant women

Table 2 shows the results of the univariate logistic regression of anaemia status (dependent variable) on some risk factors, namely age group, marital status, religion, ethnicity, occupation, gravidity, parity, blood film and gestational age of the mother. There was no significant association between anaemia status and any of the risk factors in a univariate analysis, no further multivariable logistic regression was necessary.

**Table 2:** Logistic regression of anaemia on socio-demographic characteristics in pregnant women [*N* = 409]

Variables	Anaemia status		COR (95% CI)	*p*-value
Age group	Normal n(%)	Anaemic n(%)		
40+	10 (4)	4 (3)	Ref.	
30–39	106 (39)	32 (24)	0.71 (0.22, 2.29)	0.57
20–29	127 (46)	81 (61)	1.49 (0.48, 4.66)	0.49
< 20	32 (11)	17 (12)	1.25 (0.36, 4.37)	0.72
Marital status				
Single	40 (15)	28 (21)	Ref.	
Married	235 (85)	106 (79)	0.64 (0.38, 1.10)	0.11
Religion				
Christian	235 (86)	111 (83)	Ref.	
Muslim	40 (14)	23 (17)	1.22 (0.70, 2.13)	0.49
Ethnicity				
Ewe	195 (71)	96 (72)		
Other (Guan and Akan)	80 (29)	38 (28)	0.96 (0.61, 1.52)	0.88
Occupation				
Unemployed	65 (24)	37 (28)	Ref.	
Civil servant	27 (10)	10 (8)	0.65 (0.28, 1.49)	0.31
Farming	11 (4)	4 (2)	0.63 (0.19, 2.15)	0.47
Trading	172 (62)	83 (62)	0.83 (0.50, 1.41)	0.51
Gravidity				
1–2	142 (52)	77 (58)	Ref.	
3–5	120 (44)	48 (36)	0.74 (0.48, 1.14)	
6+	13 (4)	9 (6)	1.28 (0.52, 3.12)	0.59
Parity				
None	92 (34)	52 (39)	Ref.	
1–2	116 (42.2)	52 (39)	0.79 (0.49, 1.27)	0.34
3+	67 (24.4)	30 (22)	0.79 (0.46, 1.37)	0.41
Blood film				
Negative	4 (1)	3 (2)	Ref.	
Positive	271 (99)	131 (98)	1.61 (0.39, 6.59)	0.57
Gestational Age				
1st trimester	40 (15)	18 (13)	Ref.	
2nd trimester	23 (8)	14 (11)	1.35 (0.57, 3.22)	0.49
3rd trimester	212 (77)	102 (76)	1.06 (0.58, 1.96)	0.82
Iron and folic acid supplementation during pregnancy				
No	37 (13)	19 (14)	Ref.	
Yes	238 (87)	115 (86)	0.94 (0.52, 1.71)	0.84

### Association between Anaemia and some risk factors among post-partum mothers

Table 3 shows the results of the univariate logistic regression of anaemia status on some risk factors, namely age group, marital status, religion, ethnicity, occupation, parity, gravidity and blood film of the mother. Only mothers’ age was statistically significant in the univariate analysis. [COR = 0.27 (95% CI:0.103, 0.72);0.008]. Mothers aged 20–29 were 73% less likely to be anaemic compared to those who were aged 40 + .

**Table 3:** Logistic regression of anaemia on socio-demographic characteristics in post-partum mothers [*N* = 194]

Variables	Anaemia status			
	Normal (%)	Anaemia (%)	COR (95% CI)	*p*-value
Age group				
40+	10 (6)	0 (0)	Ref.	
30–39	46 (28)	12 (38)	0.60 (0.23, 1.57)	0.30
20–29	86 (52)	10 (31)	0.27 (0.103, 0.72)	0.008*
< 20	23 (14)	10 (31)	0.11 (0.01, 1.99)	0.13
Marital status				
Single	32 (19)	9 (28)	Ref.	
Married	133 (81)	23 (72)	0.61 (0.26, 1.46)	0.27
Religion				
Christian	138 (84)	29 (91)	Ref.	
Muslim	27 (16)	3 (9)	0.53 (0.15, 1.86)	0.32
Tribe				
Ewe	125 (76)	23 (72)	Ref.	
Other (Guan and Akan)	40 (24)	9 (28)	1.22 (0.52, 2.85)	0.64
Parity				
1–2	97 (59)	21 (66)	Ref.	
3+	68 (41)	11 (34)	0.76 (0.33, 1.66)	0.49
Blood film				
Negative	163 (99)	32 (100)	Ref.	
Positive	2 (1)	0 (0)	1.01 (0.05, 21.45)	0.99
Gravidity				
1–2	97 (59)	19 (59)	Ref.	
3–5	56 (34)	10 (32)	0.91 (0.40, 2.10)	0.83
6+	12 (7)	3 (9)	1.28 (0.32, 4.96)	0.73
Estimated blood loss (*n* = 177)				
Normal	139 (94)	23 (82)	Ref.	
Abnormal	5 (3)	2 (7)	2.42 (0.44, 13.20)	0.31
PPH	5 (3)	3 (11)	3.63 (0.81, 16.22)	0.09
Iron and folic acid supplementation during pregnancy				
No	44 (27)	7 (22)	Ref.	
Yes	121 (73)	25 (78)	1.39 (0.07, 27.76)	0.83
Mosquito Net Usage				
No	102 (62)	19 (59)	Ref.	
Yes	63 (38)	13 (41)	0.89 (0.42, 1.92)	0.78

*Significant at p-valve < 0.05

## Discussion

This study examined the prevalence of anaemia and its risk factors among women attending ANC and PNC at the Hohoe Municipal Hospital, Ghana. The prevalence rate of anaemia among the ANC cohort was 33%. This is slightly lower than the 38% global anaemia prevalence in pregnant women reported by [1]. A 2018 study by Kweku et al., found anaemia prevalence among women attending ANC at the same facility to be 60% [18]. The prevalence of this study points to a situation of moderate public health problem according to WHO cut-off values for the public health significance of anaemia. Based, on their findings, it was recommended that measures be instituted to address anaemia among pregnant women in the Municipality. As such, routine iron and folic acid supplementation to pregnant women was intensified at the facility, which could have played a significant role in the low prevalence of anaemia cases recorded in this study.

Interestingly, a similar study in 2016 by Bekele, Tilahun, & Mekuria, [11] reported the same anaemia prevalence (33%) among pregnant women in Ethiopia however, those recorded in South Africa (43%) and Nigeria (55%) among the same cohort were higher [26, 27].

However, our study further revealed that, anaemia prevalence among post-partum mothers attending postnatal care clinic was 16%. This is lower than what was found in a study conducted among post-partum mothers in Ethiopia, 43% [28] and also lower than that of a study among post-partum mothers in the southern region of Madrid, 29% [29], an indication of the role iron and folic acid supplementation played as 73% of the post-partum cohort received them during pregnancy. Hence could account for the stark difference between the current study and that of [28], as no mention was made to iron and folic acid supplementation in their study. In addition, it could be due to the findings that a greater proportion of the post-partum women in our study are employed as a study by Lakew et al. in Ethiopia showed that working lactating mothers had a lower odds (AOR: 0.71; 95%CI 0.63 to 0.80) of being anaemic. In the absence of other risk factors, postnatal mothers’ age was associated with anaemia status in our study. However, a similar study by [30] found no significant association between mothers’ age and anaemia status.

The current study provides information on anaemia among post-partum mothers as currently paucity of literature exits in the Volta Region and Ghana as a whole on post-natal anaemia. This information may help in future trend analysis of anaemia among post-partum mothers to identify the burden of this condition on the vulnerable population. In the year 2003, the Ghana Health Service (GHS) in collaboration with some stakeholders rolled out a five-year integrated strategy for anaemia control in Ghana which targeted pregnant women, pre-school and school-aged children [31]. However, the vulnerable group of post-natal mothers were not targeted in this strategy for anaemia control. Post-partum mothers, who undergo some physiological losses during child birth were not target in program and this could have been as result of paucity on post-partum anaemia as at the time the program was being rolled out by the GHS.

Malaria on the other hand was less prevalent (1%) among post-partum mothers and insignificantly linked with anaemia in post-partum mothers. With fewer (62%) mothers sleeping under insecticide treated mosquito nets and the few malaria cases were recorded, this can be attributed to some form of immunity developed against malaria by postpartum women. Thus, McLean and colleagues’ study which showed a strong link between gravidity and antibodies development in improving their chances of not being anaemic [32] is evident. Therefore, the strong immunity against malaria could have been developed during pregnancy and maintained at post-partum period, fending off possible malaria attacks.

### Limitations

Since data could not be collected throughout the year, seasonality of anaemia could not be ascertained. Moreover, data on HIV/AIDS status and on dietary diversity of respondents were not collected in the study which could have had an effect on the outcomes of the study.

## Conclusion

Although the prevalence of anaemia among pregnant women found in this study was lower than the regional prevalence rate of women attending ANC (47%), it still remains unacceptably high as it points to a situation of moderate public health problem according to WHO cut-off values for the public health significance of anaemia.

Age of mother has an association with anaemia in postpartum mothers in the Hohoe municipality, as younger mothers are more likely to be anaemic than older mothers. This could be due to insufficient interaction with health care providers as a result of infrequent visits by these women because of social stigma against young mothers who are unmarried.

Strategies should therefore be put in place to encourage frequent postnatal visits by women in the younger age group. Measures must also be put in place to adopt programmes to address abnormal blood loss and PPH. This could be achieved through health education and promotion programmes. Further studies however need to be done to establish the causal effect relationship between anaemia and these risk factors in the Municipality.

## Data Availability

The datasets used or analysed during the current study are available from the corresponding author on reasonable request.

## Abbreviations

ANC: Antenatal care
COR: Crude odds ratio
ERC: Ethical Review Committee
FDH: Family health division
GHS: Ghana Health Service
Hb: Haemoglobin
ICH/GCP: International conference on Harmonization/Good Clinical Practice
PNC: Postnatal care
PPH: Postpartum haemorrhage
SPH: School of Public health
UHAS: University of Health and Allied Sciences
WBC: White blood cells
WHO: World Health Organization
